# Ethical Acceptability of Postrandomization Consent in Pragmatic Clinical Trials

**DOI:** 10.1001/jamanetworkopen.2018.6149

**Published:** 2018-12-21

**Authors:** David Gibbes Miller, Scott Y. H. Kim, Xiaobai Li, Neal W. Dickert, James Flory, Carlisle P. Runge, Clare Relton

**Affiliations:** 1 Department of Bioethics, Clinical Center, National Institutes of Health, Bethesda, Maryland; 2 Biostatistics and Clinical Epidemiology Service, Clinical Center, National Institutes of Health, Bethesda, Maryland; 3 Division of Cardiology, Department of Medicine, Emory University School of Medicine, Atlanta, Georgia; 4 Endocrinology Service, Memorial Sloan Kettering Cancer Center, New York, New York; 5 Public Health Section, School of Health and Related Research, University of Sheffield, Sheffield, United Kingdom

## Abstract

**Importance:**

Pragmatic clinical trials that seek informed consent after randomization (ie, postrandomization consent) are increasingly used, but debate on ethics persists because control arm patients are not specifically informed about the trials and randomization occurs before consent for the trials. The public’s attitude toward postrandomization consent trials is unknown, but the way the trials are described could bias people’s views.

**Objectives:**

To assess the attitudes of the US general public toward postrandomization informed consent for pragmatic trials and to measure potential framing and other factors associated with those attitudes.

**Design, Setting, and Participants:**

An online, 2 × 2 experimental survey (fielded between February 23 and April 3, 2018) portraying 4 scenarios of postrandomization informed consent (with prior broad consent for medical record use) was conducted. These scenarios included traditional randomized clinical trial language framing vs alternative framing in a high-stakes trial (ie, survival in leukemia) or low-stakes trial (ie, blood glucose level in diabetes). A total of 3793 individuals invited to participate were part of an existing panel representative of the US general public (GfK KnowledgePanel).

**Main Outcomes and Measures:**

The proportion of participants who would recommend that an ethics review board approve a postrandomization consent pragmatic trial.

**Results:**

A total of 2042 of 3739 invitees (54.6%) responded; after exclusion of 38 incomplete surveys, 2004 participants were included in the analysis. Of these, 997 (49.8%) were women, 1440 (71.9%) were white non-Hispanic, 199 (9.9%) were black non-Hispanic, and 233 (11.6%) were Hispanic. Mean (SD) age was 47.5 (17.4) years. Across scenarios, weighted data showed that 75.4% of the participants would recommend approval of the postrandomization consent pragmatic trial, 20.4% would probably not recommend approval, and 4.2% would definitely not recommend approval. Approval was not sensitive to framing language (traditional vs new framing in high-stakes scenario, 74.3% vs 76.8%, *P* = .40; in low-stakes scenario, 77.7% vs 72.9%, *P* = .10) or to the stakes (low vs high stakes in traditional framing, 77.7% vs 74.3%, *P* = .25; in new framing, 72.9% vs 76.8%, *P* = .18). Better understanding of the postrandomization consent design was associated with higher rate of approval (78.1% vs 65.0%, *P* = .002 for high-stakes scenario; 77.2% vs 64.9%, *P* = .004 for low-stakes scenario), especially among those with less education. However, opinions about personal involvement in the control arm were more cautious (range depending on scenario, 45.6%-59.7%) and sensitive to stakes but not to framing.

**Conclusions and Relevance:**

The public’s generally high rate of approval of the ethics of postrandomization informed consent for pragmatic trial designs does not appear to be affected by whether postrandomization consent design is framed using traditional randomized clinical trial terminology, regardless of the stakes of the trial. Promoting better understanding of the design may increase its acceptance by the public.

## Key Points

**Question:**

What are the opinions of the general public on the ethics of pragmatic trial designs in which specific informed consent is obtained after randomization from only the active arm of the trial?

**Findings:**

In this online survey study of 2004 members of the US general public, most participants (75.4%) would recommend approval of the ethics of the trial design, although personal involvement in such trials had a lower approval rate. Better understanding of the postrandomization consent design was associated with a higher rate of approval.

**Meaning:**

Although controversial, postrandomization consent for pragmatic trials may be ethically acceptable to the public, and education may increase its acceptance.

## Teaser

This survey study examines attitudes of the US general public toward the ethical acceptability of postrandomization informed consent in pragmatic clinical trials.

## Introduction

Pragmatic clinical trials are seen as essential to the ideal of a learning health care system that integrates medical care and clinical research to continually generate improvements in care.^1^ Such trials use inclusive criteria to broadly involve patients in health systems to produce generalizable, low-cost data and often use existing medical records as the source of outcome data.^2,3^ For nonblinded trials using usual care for the control arm, some propose obtaining consent after randomization, an idea first proposed by Zelen.^4^ Other researchers have since proposed modifications to this idea,^5,6,7,8^ and such trials are gaining increasing use.^9,10^

In postrandomization consent (PRC) trials, eligible patients are randomized to either the control arm or the intervention arm first, and then only the intervention arm participants are approached for informed consent for that trial. Those in the control arm continue their usual course of treatment and are not contacted specifically about the trial, but their medical records are used as data. Although the design features can vary,^6,8,11^ eligibility for a PRC trial can be restricted to those who have already given broad permission for the use of their medical records in research (Figure).^11^ Since in the United States this is a likely restriction,^11^ our study focused on such PRC designs.

**Figure.**
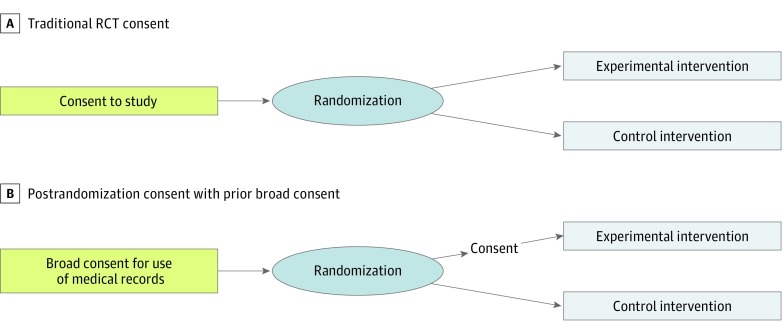
Diagram of Postrandomization Consent Process A, Traditional process for participation in randomized clinical trials (RCTs). B, Process for postrandomization consent after broad consent for use of medical records.

Postrandomization consent trials are attractive for several reasons, as they facilitate recruitment and retention of the control arm, reduce the need for resources (eg, intervention-specific informed consent is obtained from only half of the sample), reduce decisional burden for intervention arm patients (the decision is whether to accept an intervention, as in real-life settings), and potentially reduce the likelihood of disappointment for control arm patients.^8,10,11^ However, critics have argued that all patients in PRC trials, including those receiving standard treatments, are research participants who ought to go through a standard informed consent process.^12,13^ Commentators have criticized, among other things, the lack of transparency for control arm participants and the fact that a research procedure (randomization) occurs without prior consent.^14,15^

If PRC designs are to be widely used for pragmatic trials, it is vital to understand whether the public would find PRC designs ethically acceptable. Anyone who belongs to a health care system could be involved in these trials, and public trust and buy-in would be crucial to a learning health care system that uses such designs. But describing the PRC design to assess reactions to it may be challenging. For example, what is the best way to describe the control arm in a PRC design study? Traditional randomized clinical trial (RCT) language would say that those individuals are research participants of an RCT who have been assigned to the control arm of an experiment.

Presenting a study as not obtaining informed consent for participation in the RCT from such participants seems likely to bias respondents toward believing that the study is unethical. However, one could emphasize that in a PRC design none of the experiences of persons in the control arm (ie, nothing affecting their clinical care) are altered by the trial, and only their medical records are used as data (for which they would have given broad permission). These individuals serve as a kind of prospective observational cohort.^11^ Framing the study in this way runs the risk of biasing respondents toward acceptance. Thus, any study attempting to measure the public’s views on the ethical acceptability of PRC designs must take into account the potential outcome of how such designs are presented.

The purpose of this survey was to assess the US general public’s attitudes toward PRC designs (using PRC design that includes broad consent for medical record use prior to randomization) (Figure), by testing whether using a framing language different from the traditional RCT language affects the public’s views about the ethical acceptability of PRC. To increase the generalizability of our findings, we also assessed whether attitudes varied by the perceived seriousness of the disease and outcomes being studied in the PRC trials (ie, stakes). We also examined factors (comprehension of PRC design elements and demographics) associated with the public’s attitudes toward PRC.

## Methods

### Study Design

We used a 2 × 2 experimental survey design, testing the outcome of 2 different ways of describing postrandomization consent procedures (traditional RCT language framing vs new framing minimizing RCT language and noting, for example, lack of alteration of the control samples’ clinical course) and targeting disorders of differing stakes (low-stakes trial of short-term blood glucose level control in diabetes; high-stakes trial of survival rate in leukemia) on the general public’s attitudes toward PRC (eFigure and eAppendix 1 in the Supplement). This study was deemed exempt from federal research regulations by the National Institutes of Health Office of Human Subjects Research Protection.

### Setting and Participants

The survey was administered through the GfK KnowledgePanel, an online, probability-based panel representative of the adult US population. GfK follows the American Association for Public Opinion Research (AAPOR) standards for response rate reporting and our report follows the AAPOR survey disclosure checklist (eAppendix 2 in the Supplement). The GfK KnowledgePanel has previously been used for other surveys about informed consent for clinical research.^16,17,18^ Panel members are recruited through address-based sampling covering all US households, including those unreachable through random digit dialing or without internet access (internet and web-enabled devices are provided for such panel members).^19^ The survey used GfK’s Omnibus service, which fields multiple nonoverlapping surveys to a single nationally representative panel. Recruitment occurred in 2 waves between February 23 and April 3, 2018.

### Survey Measures

We conducted 3 pretest surveys on Amazon’s Mechanical Turk platform to optimize comprehension of the 4 (2 framings ×2 stakes) scenarios by soliciting open-ended feedback. The survey was modified in response to feedback from the Empirical Research Laboratory of the National Institutes of Health Clinical Center’s Department of Bioethics. The instrument was finalized after the last round of pretesting, which yielded correct answer rates ranging from 86% to 89% on the 3 comprehension questions. The participants were randomized into 1 of 4 scenarios, but all were told to assume that patients had provided prior broad consent for their medical records to be used in future research.

The traditional framing used typical RCT language (eg, participating in an RCT, experimental arm, and control arm) and a diagram showing randomization of eligible persons to 2 arms. The new framing did not use these traditional terms and instead showed some persons being randomly selected to be offered an experimental drug and the rest as continuing to receive usual care (eAppendix 1 in the Supplement).

In all 4 scenarios, the experimental intervention was described as a “commonly available nutritional supplement called MZN” that was being used as an add-on intervention to usual treatments. For the high-stakes leukemia scenario, the primary outcome was whether the drug “prolongs the lives of patients with leukemia.” For the low-stakes scenario, the primary outcome was whether MZN “lowers blood sugar more than usual care alone.”

Participants in all groups were told that an ethics review board was reviewing the proposed study to decide whether to approve it. The participants were first asked 3 knowledge questions to assess understanding of the PRC trial design and the consent process. They were then asked 3 opinion questions regarding their attitudes toward postrandomization consent: a societal perspective question about ethical acceptability (“Would you recommend to the ethics review board that they approve this study?” ie, recommendation to ethics review board question) and 2 questions from a personal perspective (“If you had diabetes and were assigned to the control group, it would mean you would not be told about the MZN study specifically. Would that be okay with you?” and “If you had diabetes and were one of the patients eligible for the MZN clinical trial, how would you feel about being randomized to 1 of the 2 groups before anyone talked with you?”). The participants were asked to provide written comments explaining their answers to the opinion questions; a separate analysis and report are planned. The wording of questions, whenever necessary, was made consistent with the scenario that the participant received. Participants were also asked to rate, on a 7-point scale, the risk level of the study proposal. GfK provided demographic information for all participants.

### Statistical Analysis

Sample size was calculated to allow us to detect an 8% difference in new and traditional framing groups’ recommendations to the ethics review board in the high-stakes scenario (assuming responses of 70% [traditional RCT framing] vs 78% [new framing]), with at least 80% power and a 2-sided α level of .05 using a χ^2^ test.

For analysis, we collapsed responses to all 3 opinion questions into dichotomous responses (eg, an overall response of yes included both probably yes and definitely yes). All analyses, unless otherwise specified, were conducted using survey weights provided by GfK, which accounted for our sample having higher educational level attainment and household income than the general population, as well as overrepresentation of persons older than 60 years and underrepresentation of black and Hispanic persons. All tests were 2 sided, with statistical significance defined as *P* < .05. Weighted proportions are reported only as percentages; unweighted proportions are reported with the number and percentages.

The primary outcome of interest was the effect of framing on the participants’ recommendations to the review board to approve or not approve the study in the high-stakes scenario; we reasoned that if a framing effect exists, it should be most detectable in such a scenario. Prespecified secondary analyses included the effect of framing on participants’ comfort with being in the control group and not knowing about the study, the effect of framing on participants’ personal acceptance (“okay”) with being randomized without being informed, the effect of stakes of the scenarios on all 3 opinion questions, and the association between knowledge question responses (using a knowledge score based on whether participants answered either 0-1 or 2-3 knowledge questions correctly) and demographics with participants’ recommendations to the ethics review board.

We used a χ^2^ test for all analyses except those involving risk level score (1-way analysis of variance of mean scores of 4 groups) and demographics. The association between demographic factors (age, sex, race/ethnicity, educational level, and income) and recommendations to the review board for the entire sample (all 4 arms combined) was examined using univariate logistic regression models. For demographic variables showing a significant association (*P* < .05) with the recommendation to the review board, we evaluated the effects of each covariate (with an additional covariate of the participants’ knowledge score) in multivariate logistic regression models. All analyses were conducted using SAS, version 9.4 (SAS Institute Inc).

## Results

### Participants

The survey was distributed to 3739 panelists, and 2042 individuals (54.6%) responded. Thirty-eight surveys that did not have responses to more than one-third of the questions were removed by GfK, leaving a total sample of 2004 participants (53.6% of surveys sent). Of the respondents, 997 (49.8%) were women, the mean (SD) age was 47.5 (17.4) years, 1440 (71.9%) were non-Hispanic white, 199 (9.9%) were non-Hispanic black, 233 (11.6%) were Hispanic, and 723 (36.1%) were college or higher graduates (unweighted demographics, Table 1).

**Table 1.** Unweighted Participant Characteristics

Characteristic	No. (%)				
	Overall (N = 2004)	High-Stakes Leukemia Scenario		Low-Stakes Diabetes Scenario	
		Traditional Framing (n = 505)	New Framing (n = 503)	Traditional Framing (n = 498)	New Framing (n = 498)
Sex					
Male	1007 (50.2)	236 (46.7)	260 (51.7)	260 (52.2)	251 (50.4)
Female	997 (49.8)	269 (53.3)	243 (48.3)	238 (47.8)	247 (49.6)
Age, y					
18-29	285 (14.2)	66 (13.1)	67 (13.3)	77 (15.5)	75 (15.1)
30-44	429 (21.4)	108 (21.4)	101 (20.1)	114 (22.9)	106 (21.3)
45-59	581 (29.0)	150 (29.7)	153 (30.4)	132 (26.5)	146 (29.3)
≥60	709 (35.4)	181 (35.8)	182 (36.2)	175 (35.1)	171 (34.3)
Race/ethnicity					
White, non-Hispanic	1440 (71.9)	362 (71.7)	357 (71.0)	372 (74.7)	349 (70.1)
Black, non-Hispanic	199 (9.9)	53 (10.5)	55 (10.9)	44 (8.8)	47 (9.4)
Other, non-Hispanic	81 (4.0)	19 (3.8)	20 (4.0)	23 (4.6)	19 (3.8)
Hispanic	233 (11.6)	57 (11.3)	60 (11.9)	54 (10.8)	62 (12.4)
≥2 Races, non-Hispanic	51 (2.5)	14 (2.8)	11 (2.2)	5 (1.0)	21 (4.2)
Education					
Less than high school	165 (8.2)	36 (7.1)	34 (6.8)	40 (8.0)	55 (11.0)
High school	526 (26.2)	139 (27.5)	133 (26.4)	125 (25.1)	129 (25.9)
Some college	590 (29.4)	156 (30.9)	154 (30.6)	133 (26.7)	147 (29.5)
College graduate or higher	723 (36.1)	174 (34.5)	182 (36.2)	200 (40.2)	167 (33.5)
Household income, $					
<25 000	268 (13.4)	68 (13.5)	51 (10.1)	70 (14.1)	79 (15.9)
25 000-<50 000	382 (19.1)	97 (19.2)	111 (22.1)	83 (16.7)	91 (18.3)
50 000-<75 000	333 (16.6)	84 (16.6)	82 (16.3)	83 (16.7)	84 (16.9)
75 000-<100 000	287 (14.3)	76 (15.0)	74 (14.7)	68 (13.7)	69 (13.9)
100 000-<125 000	252 (12.6)	57 (11.3)	67 (13.3)	60 (12.0)	68 (13.7)
125 000-<150 000	134 (6.7)	38 (7.5)	36 (7.2)	30 (6.0)	30 (6.0)
≥150 000	348 (17.4)	85 (16.8)	82 (16.3)	104 (20.9)	77 (15.5)

### Overall Attitudes Toward Postrandomization Consent Trials

The overall proportion of participants who would definitely or probably recommend that the ethics review board approve the study proposal was 75.4%, while 20.4% would probably not recommend approval, and 4.2% would definitely not recommend approval. For questions from a personal perspective, 53.2% of participants overall responded that they would be okay with being assigned to the control group in the study proposal and not being told about the study specifically. A total of 59.8% of participants overall responded that they would be okay with being a patient eligible for the study and being randomized to either the control group or the experimental group before anyone talked to them about the study. There was no significant difference in mean risk perception scores across the 4 groups (*F*_3,1987_ = 1.54, *P* = .20).

### Effect of Framing

Although we hypothesized that there would be a framing effect on participants’ recommendations to the ethics review board for the high-stakes leukemia trial scenario, no significant effect was seen: 76.8% in the new framing group would probably/definitely recommend approval vs 74.3% in the traditional framing group (*P* = .40) (Table 2). There was also no significant framing effect in the low-stakes diabetes trial scenario (72.9% would recommend approval in new framing vs 77.7% in traditional framing, *P* = .10).

**Table 2.** Effect of Framing and Stakes on Recommendation to Ethics Review Board and Personal Preferences

Response	Weighted %					χ^2^ Test (*P* Value)^b^			
	Overall (N = 2004)^a^	High-Stakes Leukemia Scenario		Low-Stakes Diabetes Scenario		Framing Effect		Stakes Effect	
		1 Traditional Framing (n = 505)	2 New Framing (n = 503)	3 Traditional Framing (n = 498)	4 New Framing (n = 498)	1 vs 2	3 vs 4	1 vs 3	2 vs 4
Probably or definitely recommend the review board approve the study	75.4	74.3	76.8	77.7	72.9	0.85 (.40)	3.01 (.10)	1.53 (.25)	2.03 (.18)
Probably or definitely okay with being assigned to control group and not being told about study	53.2	49.2	45.6	58.4	59.7	1.34 (.27)	0.19 (.68)	8.39 (.006)	20.10 (<.001)
Probably or definitely okay with being randomized without being informed	59.8	52.1	59.8	60.6	66.8	6.01 (.02)	4.10 (.05)	7.32 (.01)	5.26 (.03)

^a^ Each test excluded cases that did not respond to the corresponding question.

^b^ All tests used 1 *df*. All analyses were weighted.

Regarding whether participants would be okay with being assigned to the control group without being told about the study, there was no significant difference between traditional and new framing in the high-stakes group (49.2% vs 45.6%, *P* = .27) or in the low-stakes group (58.4% vs 59.7%, *P* = .68). Most participants also stated they would be okay with being randomized without being informed, but this response was subject to a small framing effect that was significant in the high-stakes scenario (52.1% traditional framing vs 59.8% new framing, *P* = .02) and marginal in the low-stakes scenario (60.6% in traditional framing vs 66.8% in new framing, *P* = .05).

### Effect of Stakes

The stakes of the study proposal had no effect on whether participants would recommend that the ethics review board approve the study (77.7% in the low-stakes scenario vs 74.3% in the high-stakes scenario, *P* = .25 for traditional framing; 72.9% in the low-stakes scenario vs 76.8% in the high-stakes scenario, *P* = .18 for new framing).

The stakes of the study proposal had a significant effect for both questions about personal involvement. The proportion of participants responding that they would be okay with being in the control group and not being told about the study was 58.4% in the low-stakes and 49.2% in the high-stakes scenarios (*P* = .006) with traditional framing and 59.7% in the low-stakes and 45.6% in the high-stakes scenarios (*P* < .001) with new framing. The proportion of participants who would be okay with being randomized for the study without being informed was 60.6% in the low-stakes vs 52.1% in the high-stakes scenarios (*P* = .01) for traditional framing and 66.8% vs 59.8% for new framing (*P* = .03).

### Association With Knowledge Scores

Overall, 1701 participants (86.4%) answered 2 or more knowledge questions correctly (703 [35.7%] answered 2 questions correctly and 998 [50.7%] answered all 3 questions correctly). There was no significant difference in the number of knowledge questions answered correctly between the high- and low-stakes scenario groups (86.8% high stakes vs 85.3% low stakes, *P* = .34) or between the traditional and new framing groups (85.2% vs 86.9%, *P* = .30).

Answering 2 or more knowledge questions correctly was associated with increased acceptance of the PRC study design. In both the high- and low-stakes groups, those who understood the study proposal were more likely to recommend that the ethics review board approve the study (77.2% vs 64.9%, *P* = .004 in low-stakes and 78.1% vs 65.0%, *P* = .002 in high-stakes scenarios) (Table 3). This association was also seen among those who were okay with being in the control group of the study and not being told about the study (61.7% vs 41.6%, *P* < .001 in low-stakes and 49.5% vs 39.5%, *P* = .04 in high-stakes scenarios), and among those who were okay with being randomized without being informed (67.1% vs 44.6%, *P* < .001 in low-stakes and 58.8% vs 43.2%, *P* = .001 in high-stakes scenarios).

**Table 3.** Association Between Knowledge Question Responses and Recommendation to Ethics Review Board and Personal Preferences

Response	Knowledge Questions Correct, %^a^							
	Overall (N = 2004)		High-Stakes Leukemia Scenario (n = 1008)^b^			Low-Stakes Diabetes Scenario (n = 996)^b^		
	0-1 (n = 268)	2-3 (n = 1701)	0-1 (n = 139)	2-3 (n = 851)	χ^2^ (*P* Value)^c^	0-1 (n = 129)	2-3 (n = 850)	χ^2^ (*P* Value)^c^
Recommends that the review board approve the study	65.0	77.6	65.0	78.1	11.55 (.002)	64.9	77.2	9.08 (.004)
Okay with being assigned to control group and not being told about study	40.5	55.6	39.5	49.5	4.85 (.04)	41.6	61.7	18.46 (<.001)
Okay with being randomized without being informed	43.8	62.9	43.2	58.8	12.12 (.001)	44.6	67.1	23.90 (<.001)

^a^ Each test excluded cases that did not respond to the corresponding questions. The 35 cases that did not include a response to at least 1 knowledge question were excluded (18 questions in high-stakes groups and 17 in low-stakes groups).

^b^ Both new and traditional framing groups were collapsed into a single group for this analysis.

^c^ All tests used 1 *df*. All analyses were weighted.

### Demographic Factors and Recommendation to Ethics Review Board

In unadjusted analysis, race/ethnicity, educational level, and income, but not age and sex, were significantly associated with participants’ recommendations to the ethics review board (Table 4). Black participants were less likely than white, non-Hispanic participants to recommend that the review board approve the study. A multivariate logistic model with all significant covariates showed that only educational level (less than high school as reference: high school graduate, odds ratio [OR], 1.62; 95% CI, 1.08-2.43; some college, OR, 1.50; 95% CI, 1.00-2.25; college graduate or higher, OR, 1.82; 95% CI, 1.18-2.80) and knowledge score (≥2 correct vs not, OR, 1.80; 95% CI, 1.34-2.41) had significant independent associations. In a model with educational level (dichotomized as less than high school vs high school graduate or higher, because of similar ORs in the above model), knowledge score, and a term for interaction of the 2 models, we found a significant interaction between knowledge score and educational level such that the associations with knowledge score in the lowest educational level group (less than a high school education) were greater than in those with more than a high school education (OR, 6.69; 95% CI, 2.49-17.96 vs OR, 1.56; 95% CI, 1.13-2.14; *P* = .006).

**Table 4.** Association Between Demographics and Knowledge Scores With Recommendation to Review Board for All 4 Groups^a^

Characteristic	Overall Unweighted n = 2004), No. (%)	Recommending Approval, Weighted %	χ^2^ (*P* Value)^b^
Sex			
Male	1007 (50.2)	75.3	0.016 (.91)
Female	997 (49.8)	75.5	
Income category, $			
<25 000	268 (13.4)	66.4	21.40 (.004)
25 000-<50 000	382 (19.1)	73.0	
50 000-<75 000	333 (16.6)	77.6	
75 000-<100 000	287 (14.3)	79.8	
100 000-<125 000	252 (12.6)	77.9	
125 000-<150 000	134 (6.7)	81.5	
≥150 000	348 (17.4)	76.6	
Education level			
<High school	165 (8.2)	63.3	23.05 (<.001)
High school graduate	526 (26.2)	75.0	
Some college	590 (29.4)	75.5	
≥College graduate	723 (36.1)	79.8	
Race/ethnicity			
White, non-Hispanic	1440 (71.9)	77.9	14.77 (.03)
Black, non-Hispanic	199 (9.9)	67.3	
Other, Non-Hispanic	81 (4.0)	72.8	
Hispanic	233 (11.6)	72.0	
≥2 Races, non-Hispanic	51 (2.5)	76.5	
Age, y			
18-24	184 (9.2)	68.8	10.08 (.17)
25-34	401 (20.0)	72.0	
35-44	329 (16.4)	78.1	
45-54	316 (15.8)	76.2	
55-64	396 (19.8)	78.0	
65-74	259 (12.9)	76.0	
≥75	119 (5.9)	77.7	
Knowledge questions correct			
0-1	268 (13.6)	65.0	20.53 (<.001)
2-3	1701 (86.4)	77.6	

^a^ Incomplete responses in 35 participants.

^b^ χ^2^ Test between demographic category and percentage recommending approval.

## Discussion

Although pragmatic RCTs using PRC have potential advantages, commentators continue to debate the ethical acceptability of the lack of specific consent from the control group and of randomization (a research procedure) before consent for such RCTs.^11,12^ When ethical intuition is divided in a policy area that requires the support of the public (as pragmatic trials must be), it is important to understand the public’s stance regarding the ethics of these issues.

Our primary outcome (framing association in the high-stakes scenario) was based on the concern that the language of traditional RCTs, if used to describe elements of PRC designs, could lead respondents to not recognize important ethical features of PRC studies. We found, however, that the participants’ recommendations to an ethics review board were not affected by how the PRC was described. In light of this information, it appears that the concern over the influence of language may loom large for ethicists and institutional review board members who are immersed in the ethics debates but that the public are able to focus on design elements of PRC trials without being influenced by the language.

This study yielded several additional findings. First, there was significant support generally for postrandomization consent in pragmatic trials, with 75.4% of participants recommending that the ethics review board approve the study and 4.2% of people saying they would definitely not recommend approval.

Second, the questions about personal involvement in the trial yielded lower—but still more than half—approval rates (53.2% for being in the control group without being told about the study and 59.8% for being randomized without being informed). We do not think this discrepancy between perspectives taken is irrational (eg, one might find research studies ethically acceptable but may not personally wish to participate, indicating that many people may find lack of full transparency involved in the control arm of PRC trials personally unacceptable). The results echo a previous finding that, although 68% of US adults are willing to provide open-ended consent for research with donated tissues, fewer are willing when told about potential uses under such open-ended consent (eg, 55% for use in commercial drug development).^17^ However, these respondents’ preferred solution was not specific consent for each research use but some form of enhanced broad consent, such as broad consent with a general caution (eg, some people might have moral, religious, or cultural concerns about some uses) or broad consent with easy access to a list of research projects with the option to withdraw.^17^

Third, most people understood (answered 2-3 knowledge questions correctly) the key design elements of the described PRC trials and they were significantly more likely to approve of a PRC design than those with poor comprehension. This association was not fully explained by any demographic variable, including educational level. The interaction (this knowledge association was most pronounced in the lowest educational level group) also highlights the importance of comprehending the rather complex design of PRC studies. Overall, the results suggest that public education will help improve acceptance of PRC in pragmatic trials.

### Limitations

There are several limitations to this study. First, this survey used hypothetical scenarios, asking participants to play roles with which they may not be familiar (albeit as a tool for gauging their intuition on ethics). Second, this study tested a type of PRC design that requires broad permission for the use of patients’ medical records—a feature that is not shared by all PRC designs.^5,6^ Third, although a 55% response rate is good for an online public survey, we had to use weighted analyses to optimize generalizability. Fourth, the RCT scenarios portrayed a commonly used food supplement as the intervention, which may not generalize to scenarios of riskier interventions. We can only speculate on how a riskier intervention might affect attitudes since such an intervention that would be reasonable to test should also have at least a perceived higher potential for benefit.

## Conclusions

As pragmatic clinical trials using novel consent designs become more common, understanding the public’s attitudes toward the ethical dimensions of such trials may help researchers design and institutional review boards evaluate PRC protocols in a more informed manner. We found a generally high rate of acceptability regarding the ethics of PRC trials that was not sensitive to whether traditional RCT language is used. Still, the high level of approval must be interpreted in light of a lower, but still relatively high, level of comfort in situations with more affective salience for the participants (ie, when they are asked to imagine themselves in the PRC trials), especially in higher-stakes trials. A key finding was that participants who better understood PRC design were more likely to approve of the PRC study, indicating the importance of educating people about such designs. As pragmatic trials grow in popularity, greater public education may be an important step to ensuring transparency, trust, and acceptance of the clinical research enterprise in general and the PRC designs in particular.

## Notes

Supplement. **eFigure.** Study Flow Design **eAppendix 1.** Post-Randomization Consent Survey Instrument **eAppendix 2.** AAPOR Survey Disclosure Checklist and Gfk Methodology

## References

[1] Olsen L, Aisner D, McGinnis J *The Learning Healthcare System* Washington, DC: Institute of Medicine, National Academies; 2007. 21452449

[2] Tunis SR, Stryer DB, Clancy CM Practical clinical trials: increasing the value of clinical research for decision making in clinical and health policy. JAMA. 2003;290(12):-. doi:10.1001/jama.290.12.1624 14506122

[3] Ford I, Norrie J Pragmatic trials. N Engl J Med. 2016;375(5):454-463. doi:10.1056/NEJMra1510059 27518663

[4] Zelen M A new design for randomized clinical trials. N Engl J Med. 1979;300(22):1242-1245. doi:10.1056/NEJM197905313002203 431682

[5] Flory JH, Mushlin AI, Goodman ZI Proposals to conduct randomized controlled trials without informed consent: a narrative review. J Gen Intern Med. 2016;31(12):1511-1518. doi:10.1007/s11606-016-3780-5 27384536 PMC5130947

[6] Relton C, Torgerson D, O’Cathain A, Nicholl J Rethinking pragmatic randomised controlled trials: introducing the “cohort multiple randomised controlled trial” design. BMJ. 2010;340:c1066. doi:10.1136/bmj.c1066 20304934

[7] Relton C, Burbach M, Collett C, The ethics of “trials within cohorts” (TwiCs): 2nd international symposium. Trials. 2017;18(suppl 2):244. doi:10.1186/s13063-017-1961-0

[8] Vickers AJ, Young-Afat DA, Ehdaie B, Kim SYH Just-in-time consent: the ethical case for an alternative to traditional informed consent in randomized trials comparing an experimental intervention with usual care. Clin Trials. 2018;15(1):3-8. 29224379 10.1177/1740774517746610 PMC5799028

[9] Relton C, Thomas K, Nicholl J, Uher R Review of an innovative approach to practical trials: the “cohort multiple RCT” design. Trials. 2015;16(suppl 2):114-P114. doi:10.1186/1745-6215-16-S2-P11425873255

[10] Simon GE, Beck A, Rossom R, Population-based outreach versus care as usual to prevent suicide attempt: study protocol for a randomized controlled trial. Trials. 2016;17(1):452. doi:10.1186/s13063-016-1566-z 27634417 PMC5025595

[11] Kim SYH, Flory J, Relton C Ethics and practice of trials within cohorts: an emerging pragmatic trial design. Clin Trials. 2018;15(1):9-16. doi:10.1177/1740774517746620 29224380 PMC6006508

[12] Weijer C, Goldstein CE, Taljaard M TwiC or treat? are trials within cohorts ethically defensible? Clin Trials. 2018;15(1):21-24. doi:10.1177/1740774517746622 29250989 PMC5802521

[13] Hawkins JS The ethics of Zelen consent. J Thromb Haemost. 2004;2(6):882-883. doi:10.1111/j.1538-7836.2004.00782.x 15140121

[14] Wendler D Innovative approaches to informed consent for randomized clinical trials: identifying the ethical challenges. Clin Trials. 2018;15(1):17-20. doi:10.1177/1740774517746621 29250988 PMC5799024

[15] Ellenberg SS Randomization designs in comparative clinical trials. N Engl J Med. 1984;310(21):1404-1408. doi:10.1056/NEJM198405243102141 6717522

[16] Nayak RK, Wendler D, Miller FG, Kim SYH Pragmatic randomized trials without standard informed consent? a national survey. Ann Intern Med. 2015;163(5):356-364. doi:10.7326/M15-0817 26215125 PMC5573142

[17] Tomlinson T, De Vries R, Ryan K, Kim HM, Lehpamer N, Kim SYH Moral concerns and the willingness to donate to a research biobank. JAMA. 2015;313(4):417-419. doi:10.1001/jama.2014.16363 25626040 PMC4443895

[18] Dickert NW, Wendler D, Devireddy CM, Consent for pragmatic trials in acute myocardial infarction. J Am Coll Cardiol. 2018;71(9):1051-1053. doi:10.1016/j.jacc.2017.12.043 29495987 PMC6151267

[19] GfK KnowledgePanel: a methodological overview. http://www.gfk.com/fileadmin/user_upload/dyna_content/US/documents/KnowledgePanel_-_A_Methodological_Overview.pdf. Accessed May 8, 2018.

