# Supplementary material for: Ethical Acceptability of Postrandomization Consent in Pragmatic Clinical Trials
Source: JAMA Netw Open. 2018 Dec 21;1(8):e186149. doi: 10.1001/jamanetworkopen.2018.6149 (PMC6324565; doi:10.1001/jamanetworkopen.2018.6149)
Supplement: Supplement. — eFigure. Study Flow Design eAppendix 1. Post-Randomization Consent Survey Instrument eAppendix 2. AAPOR Survey Disclosure Checklist and Gfk Methodology [file jamanetwopen-1-e186149-s001.pdf]

Supplementary Online Content

Miller DG, Kim SYH, Li X, et al. Ethical acceptability of postrandomization consent in pragmatic clinical trials. *JAMA Netw Open*. 2018;1(8): e186149.
doi:10.1001/jamanetworkopen.2018.6149

eFigure. Study Flow Design

eAppendix 1. Post-Randomization Consent Survey Instrument

eAppendix 2. AAPOR Survey Disclosure Checklist and Gfk Methodology

This supplementary material has been provided by the authors to give readers additional information about their work.

© 2018 Miller DG et al. *JAMA Network Open*

eFigure. Study Flow Design

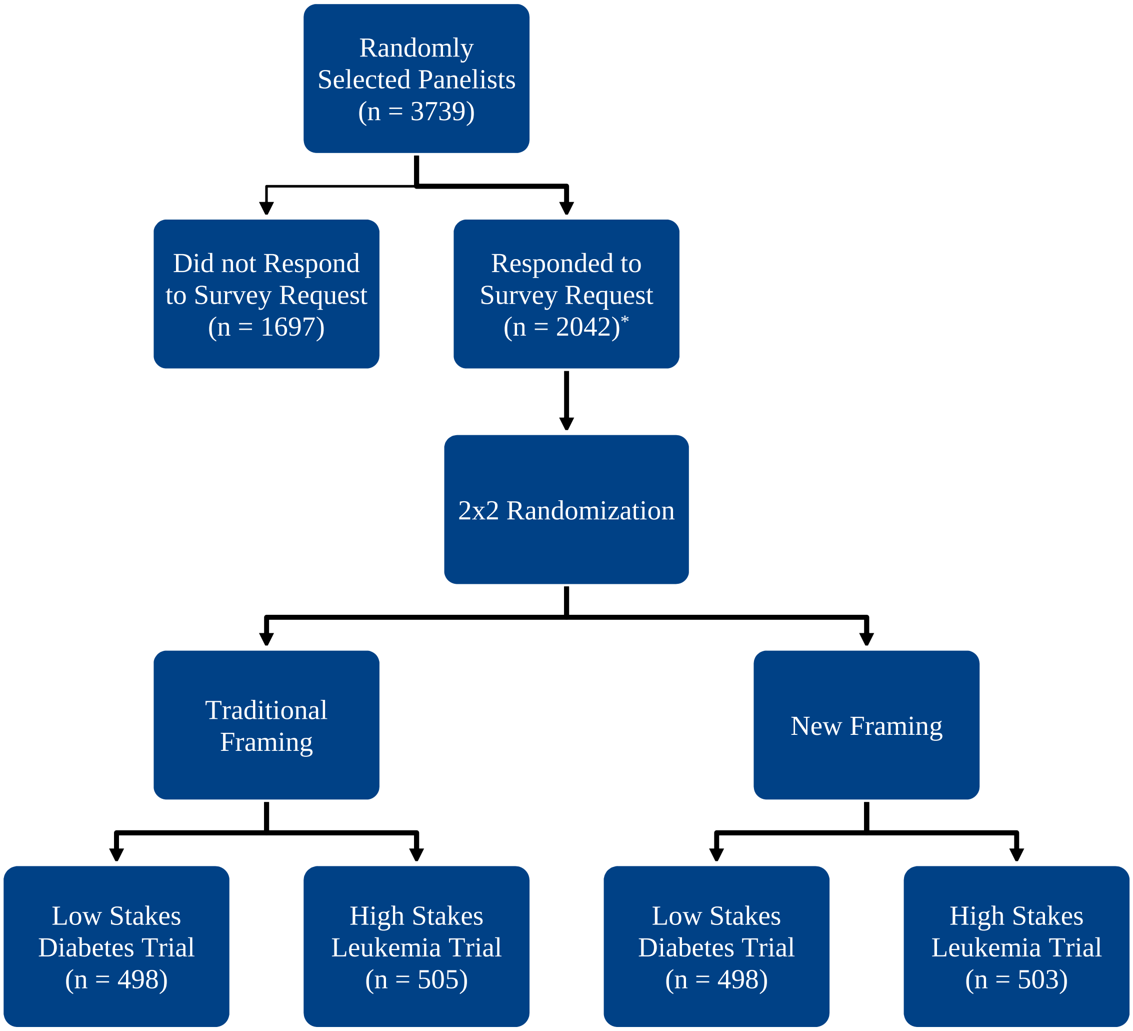

*Sum of four arms do not add to 2042 because 38 participants who did not complete more than 1/3 of questions were removed by GfK.

eAppendix 1. Post-Randomization Consent Survey Instrument

[The survey below uses a hypothetical RCT with several pragmatic features. It describes a very inclusive recruitment plan of ‘real life’ patients—all patients in a health care system with diabetes or leukemia, depending on the scenario—and the use of medical records as sources of outcome, as well as using the clinic itself as the setting for the RCT.]

[All Respondents]

In this next set of questions, we are trying to learn what type of permission is needed from patients when they are asked to participate in different types of medical research.

We will describe one type of research using a hypothetical (pretend) scenario and ask your opinions about it.

[Randomize respondents to 4 even groups]

[Group 1: Low Stakes, Traditional Framing]

Diabetes
Diabetes is a chronic medical condition in which a patient’s blood sugar levels are too high. Usual care for patients with diabetes involves blood sugar monitoring, encouraging healthy eating, and sometimes medicines (like insulin). Patients can manage diabetes and live for a long time with these usual care interventions.

Now imagine that there is a commonly available nutritional supplement called MZN. Researchers recently discovered that in animals with diabetes, MZN might help to control the disease. Although people take MZN for other reasons, doctors do not normally talk to their diabetic patients about MZN because no one knows whether it will help lower blood sugar in humans with diabetes. So the researchers want to conduct a study—called a randomized clinical trial—to find out whether **taking MZN in addition to usual care** lowers blood sugar more than **usual care alone**.

Prior Permission for Future Use of Medical Records Will Be Obtained
The following study will take place in an academic healthcare system involving only diabetic patients who have already agreed to have their medical records used in future research studies. All research with these medical records is reviewed by an ethics review board, and the records are kept safe and confidential.

Research Proposal
Over the next five screens, we will describe the steps researchers will use to conduct a randomized clinical trial of MZN.

---

1. Identify all patients in the healthcare system who have diabetes.

2. Randomize (like flipping a coin) these patients to the control group or the experimental group.

3A. The experimental group will be asked if they are willing to take MZN in addition to usual care as part of the clinical trial. They will be told about the purpose of the trial, the potential consequences of taking MZN, and other details about the clinical trial. They will be free to refuse the MZN.

3B. The control group will continue to receive usual care for diabetes. They will not be told about the MZN clinical trial. But their medical records will be used as data.

4. At the end of the randomized clinical trial, the researchers will compare the average blood sugar levels between the two groups using their medical records.

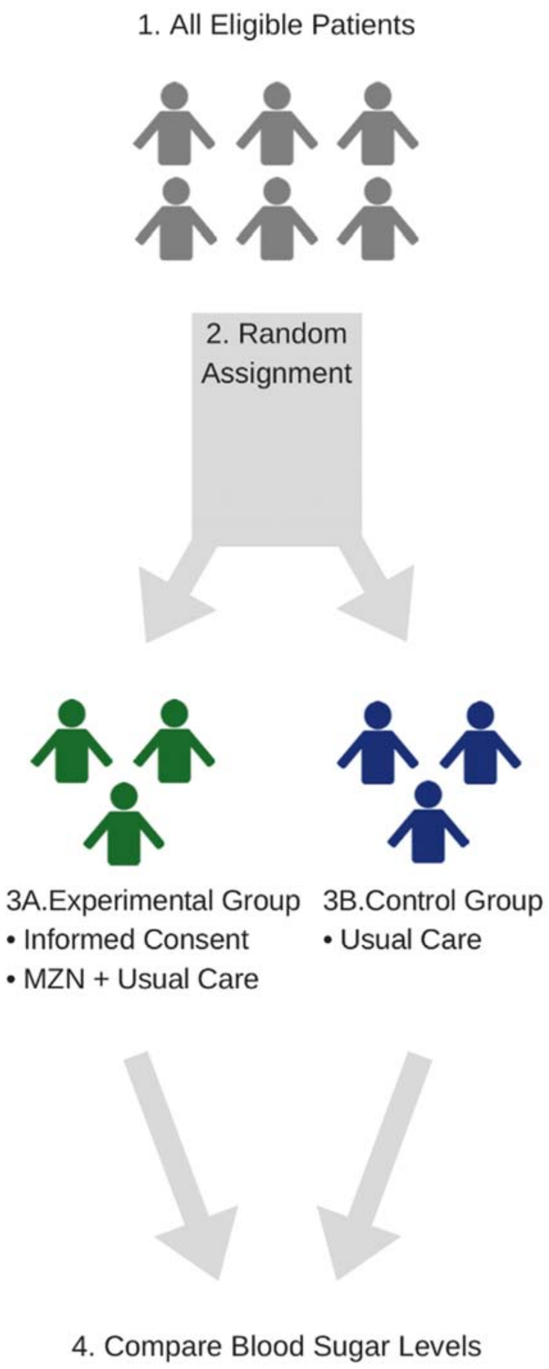

Ethics Review Board
An ethics review board is reviewing the study to decide whether to approve it.

Some members of the review board point out that patients in the control group will not know that they are participating in the MZN clinical trial. Those patients also will not know that other people were randomized to the experimental group.

Other members think that the control group does not need to know that they are participating in the MZN clinical trial since nothing about their usual care for diabetes will change, and all of the patients already provided permission for the use of their medical records in future research studies.

Now we are going to ask you some questions about the MZN clinical trial.

Q01a
Will the patients in the control group be told that the MZN study is being conducted?

1. Yes
2. No

Q02a
Will the patients in the experimental group have an opportunity to refuse to take MZN?

1. Yes
2. No

Q03a
Patients in the MZN clinical trial are randomized to one of two groups before any of them are told about the MZN clinical trial.

1. True
2. False

Q04a
Would you recommend to the ethics review board that they approve this study?

1. Definitely yes
2. Probably yes
3. Probably not
4. Definitely not

Q04a_b Please briefly explain why you chose that answer.
[text box]

Q05a
If you had diabetes and were assigned to the control group, it would mean you would not be told about the MZN study specifically. Would that be okay with you?

1. Definitely okay
2. Probably okay
3. Probably not okay
4. Definitely not okay

Q05a_b Please briefly explain why you chose that answer.
[text box]

Q06a

If you had diabetes and were one of the patients eligible for the MZN clinical trial, how would you feel about being randomized to one of the two groups before anyone talked with you?

1. Definitely okay
2. Probably okay
3. Probably not okay
4. Definitely not okay

Q06a_b Please briefly explain why you chose that answer.
[text box]

Q07a
In your opinion, how risky is it for the patients to be part of the MZN study overall?

1. Not at all risky
2.
3.
4.
5.
6.
7. Extremely risky

[Group 2: Low Stakes, New Framing]
Diabetes
Diabetes is a chronic medical condition in which a patient's blood sugar levels are too high. Usual care for patients with diabetes involves blood sugar monitoring, encouraging healthy eating, and sometimes medicines (like insulin). Patients can manage diabetes and live for a long time with these usual care interventions.

Now imagine that there is a commonly available nutritional supplement called MZN. Researchers recently discovered that in animals with diabetes, MZN might help to control the disease. Although people take MZN for other reasons, doctors do not normally talk to their diabetic patients about MZN because no one knows whether it will help lower blood sugar in humans with diabetes. So the researchers want to conduct a study to find out whether **taking MZN in addition to usual care** lowers blood sugar more than **usual care alone**.

Prior Permission for Future Use of Medical Records Will Be Obtained
The following study will take place in an academic healthcare system involving only diabetic patients who have already agreed to have their medical records used in future research studies. All research with these medical records is reviewed by an ethics review board, and the records are kept safe and confidential.

Research Proposal
Over the next four screens, we will describe the steps researchers will use to determine if MZN in addition to usual care is better than usual care alone.

---

1. Identify all patients in the healthcare system who have diabetes.

2. Randomly select (like flipping a coin) about half of the patients and ask them if they are willing to take MZN in addition to usual care as part of the study. They will be told about the purpose of the research, the potential consequences of taking MZN, and other details about the study. They will be free to refuse the MZN.

3. Everyone else will continue to receive usual care for diabetes. They will not be told about the MZN study. But their medical records will be used as data.

4. At the end of the study period, the researchers will compare the average blood sugar levels between the two groups using their medical records.

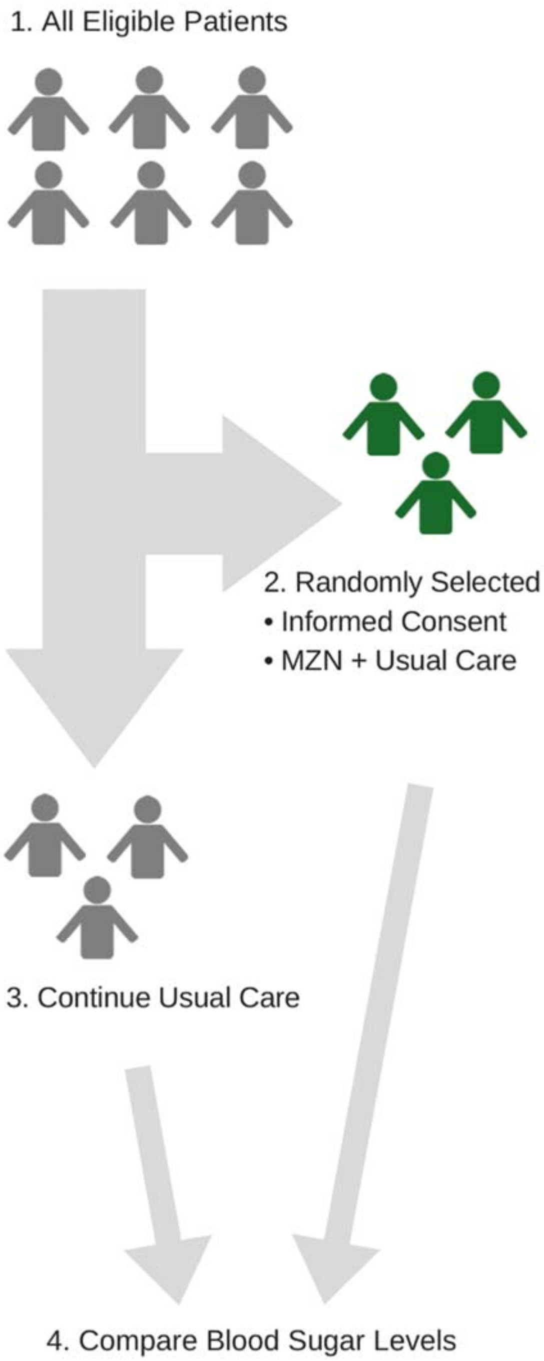

Ethics Review Board
An ethics review board is reviewing the study to decide whether to approve it.

Some members of the review board point out that patients who are not selected for MZN will not know that their medical records are being used in the MZN study specifically. Those patients also will not know that other people were randomly offered MZN.

Other members think that those not selected for MZN do not need to know about the MZN study since nothing about their usual care for diabetes will change, and all of the patients already provided permission for the use of their medical records in future research studies.

Now we are going to ask you some questions about the MZN study.

Q01b
Will patients who are not offered MZN be told that the MZN study is being conducted?

1. Yes
2. No

Q02b
Will the patients who are offered MZN have an opportunity to refuse to take MZN?

1. Yes
2. No

Q03b
Some patients are randomly selected to be offered MZN before any patients are told about the MZN study.

1. True
2. False

Q04b
Would you recommend to the ethics review board that they approve this study?

1. Definitely yes
2. Probably yes
3. Probably not
4. Definitely not

Q04b_b Please briefly explain why you chose that answer.
[text box]

Q05b
If you had diabetes and were not randomly selected for MZN, it would mean you would not be told about the MZN study specifically. Would that be okay with you?

1. Definitely okay
2. Probably okay
3. Probably not okay
4. Definitely not okay

Q05b_b Please briefly explain why you chose that answer.
[text box]

Q06b

If you had diabetes and were one of the patients eligible for the MZN study, how would you feel about the random selection (for whether to be offered MZN) taking place before anyone talked with you?

1. Definitely okay
2. Probably okay
3. Probably not okay
4. Definitely not okay

Q06b_b Please briefly explain why you chose that answer.
[text box]

Q07b
In your opinion, how risky is it for the patients to be part of the MZN study overall?

1. Not at all risky
2.
3.
4.
5.
6.
7. Extremely risky

[Group 3: High Stakes, Traditional Framing]
Leukemia
Leukemia is an aggressive cancer of the blood cells. Usual treatment for leukemia is chemotherapy and sometimes a bone marrow transplant. Patients may die from leukemia if these treatments do not work.

Now imagine that there is a commonly available nutritional supplement called MZN. Researchers recently discovered that in animals with leukemia, MZN might help to treat the cancer. Although people take MZN for other reasons, doctors do not normally talk to their leukemia patients about MZN because no one knows whether it will help improve survival in humans with leukemia. So the researchers want to conduct a study—called a randomized clinical trial—to find out whether **taking MZN in addition to usual treatment** prolongs the lives of patients with leukemia more than **usual treatment alone**.

Prior Permission for Future Use of Medical Records Will Be Obtained
The following study will take place in an academic healthcare system involving only leukemia patients who have already agreed to have their medical records used in future research studies. All research with these medical records is reviewed by an ethics review board, and the records are kept safe and confidential.

Research Proposal
Over the next five screens, we will describe the steps researchers will use to conduct a randomized clinical trial of MZN.

---

1. Identify all patients in the healthcare system who have leukemia.

2. Randomize (like flipping a coin) these patients to the control group or the experimental group.

3A. The experimental group will be asked if they are willing to take MZN in addition to usual treatment as part of the clinical trial. They will be told about the purpose of the trial, the potential consequences of taking MZN, and other details about the clinical trial. They will be free to refuse the MZN.

3B. The control group will continue the usual course of treatment for leukemia. They will not be told about the MZN clinical trial. But their medical records will be used as data.

4. At the end of the randomized clinical trial, the researchers will compare survival rates between the two groups using their medical records.

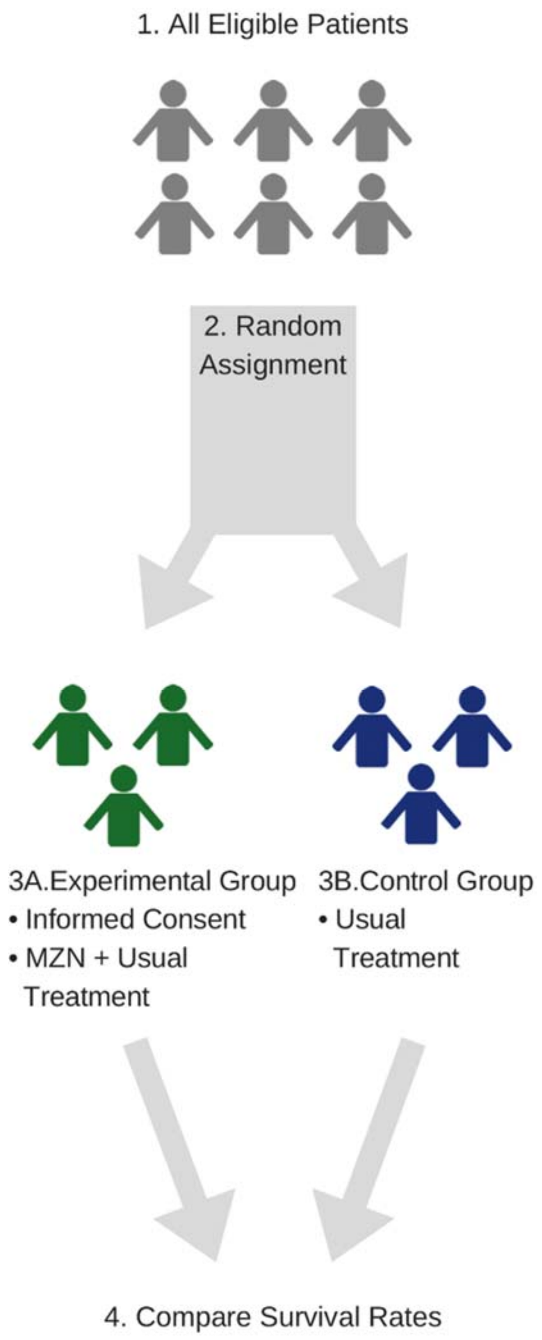

Ethics Review Board
An ethics review board is reviewing the study to decide whether to approve it.

Some members of the review board point out that patients in the control group will not know that they are participating in the MZN clinical trial. Those patients also will not know that other people were randomized to the experimental group.

Other members think that the control group does not need to know that they are participating in the MZN clinical trial since nothing about their usual treatment for leukemia will change, and all of the patients already provided permission for the use of their medical records in future research studies.

Now we are going to ask you some questions about the MZN clinical trial.

Q01c
Will the patients in the control group be told that the MZN study is being conducted?

1. Yes
2. No

Q02c
Will the patients in the experimental group have an opportunity to refuse to take MZN?

1. Yes
2. No

Q03c
Patients in the MZN clinical trial are randomized to one of two groups before any of them are told about the MZN clinical trial.

1. True
2. False

Q04c
Would you recommend to the ethics review board that they approve this study?

1. Definitely yes
2. Probably yes
3. Probably not
4. Definitely not

Q04c_b Please briefly explain why you chose that answer.
[text box]

Q05c
If you had leukemia and were assigned to the control group, it would mean you would not be told about the MZN study specifically. Would that be okay with you?

1. Definitely okay
2. Probably okay
3. Probably not okay
4. Definitely not okay

Q05c_b Please briefly explain why you chose that answer.
[text box]

Q06c

If you had leukemia and were one of the patients eligible for the MZN clinical trial, how would you feel about being randomized to one of the two groups before anyone talked with you?

1. Definitely okay
2. Probably okay
3. Probably not okay
4. Definitely not okay

Q06c_b Please briefly explain why you chose that answer.
[text box]

Q07c
In your opinion, how risky is it for the patients to be part of the MZN study overall?

1. Not at all risky
2.
3.
4.
5.
6.
7. Extremely risky

[Group 4: High Stakes, New Framing]
Leukemia
Leukemia is an aggressive cancer of the blood cells. Usual treatment for leukemia is chemotherapy and sometimes a bone marrow transplant. Patients may die from leukemia if these treatments do not work.

Now imagine that there is a commonly available nutritional supplement called MZN. Researchers recently discovered that in animals with leukemia, MZN might help to treat the cancer. Although people take MZN for other reasons, doctors do not normally talk to their leukemia patients about MZN because no one knows whether it will help improve survival in humans with leukemia. So the researchers want to conduct a study to find out whether **taking MZN in addition to usual treatment** prolongs the lives of patients with leukemia more than **usual treatment alone.**

Prior Permission for Future Use of Medical Records Will Be Obtained
The following study will take place in an academic healthcare system involving only leukemia patients who have already agreed to have their medical records used in future research studies. All research with these medical records is reviewed by an ethics review board, and the records are kept safe and confidential.

Research Proposal
Over the next four screens, we will describe the steps researchers will use to determine if MZN in addition to usual care is better than usual care alone.

1. Identify all patients in the healthcare system who have leukemia.

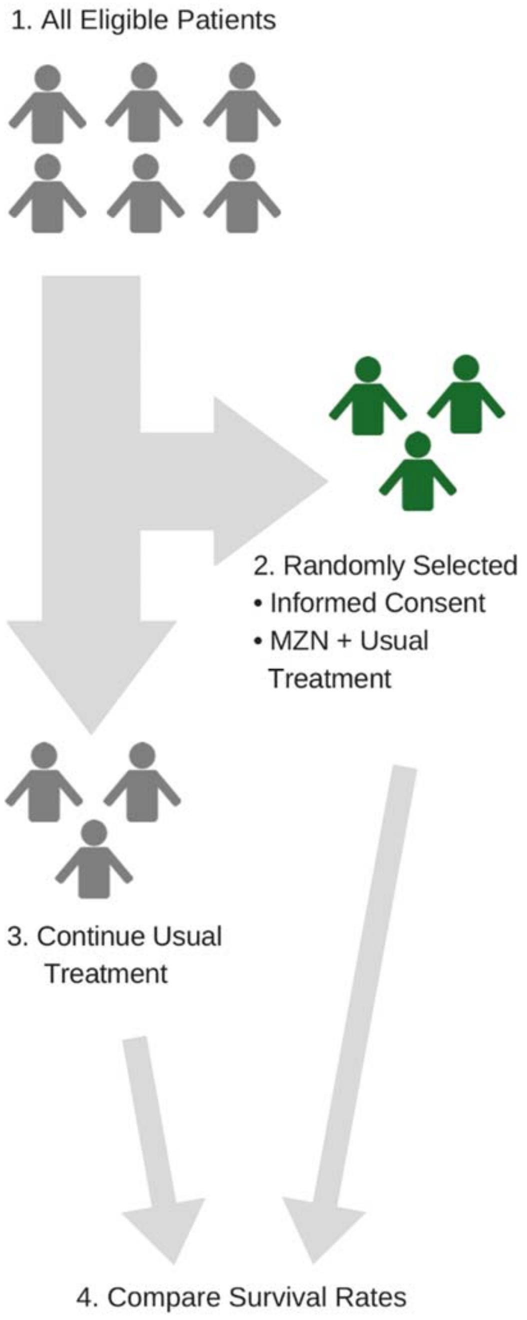

2. Randomly select (like flipping a coin) about half of the patients and ask them if they are willing to take MZN in addition to usual treatment as part of the study. They will be told about the purpose of the research, the potential consequences of taking MZN, and other details about the study. They will be free to refuse the MZN.

3. Everyone else will continue the usual course of treatment for leukemia. They will not be told about the MZN study. But their medical records will be used as data.

4. At the end of the study period, the researchers will compare survival rates between the two groups using their medical records.

Ethics Review Board
An ethics review board is reviewing the study to decide whether to approve it.

Some members of the review board point out that patients who are not selected for MZN will not know that their medical records are being used in the MZN study specifically. Those patients also will not know that other people were randomly offered MZN.

Other members think that those not selected for MZN do not need to know about the MZN study since nothing about their usual treatment for leukemia will change, and all of the patients already provided permission for the use of their medical records in future research studies.

Now we are going to ask you some questions about the MZN study.

Q01d
Will patients who are not offered MZN be told that the MZN study is being conducted?

1. Yes
2. No

Q02d
Will the patients who are offered MZN have an opportunity to refuse to take MZN?

1. Yes
2. No

Q03d
Some patients are randomly selected to be offered MZN before any patients are told about the MZN study.

1. True
2. False

Q04d
Would you recommend to the ethics review board that they approve this study?

1. Definitely yes
2. Probably yes
3. Probably not
4. Definitely not

Q04d_b Please briefly explain why you chose that answer
[text box]

Q05d
If you had leukemia and were not randomly selected for MZN, it would mean you would not be told about the MZN study specifically. Would that be okay with you?

1. Definitely okay
2. Probably okay
3. Probably not okay
4. Definitely not okay

Q05d_ b Please briefly explain why you chose that answer
[text box]

Q06d

If you had leukemia and were one of the patients eligible for the MZN study, how would you feel about the random selection (for whether to be offered MZN) taking place before anyone talked with you?

1. Definitely okay
2. Probably okay
3. Probably not okay
4. Definitely not okay

Q06d_b Please briefly explain why you chose that answer
[text box]

Q07d
In your opinion, how risky is it for the patients to be part of the MZN study overall?

1. Not at all risky
2.
3.
4.
5.
6.
7. Extremely risky

eAppendix 2. AAPOR Survey Disclosure Checklist and Gfk Methodology

In accordance with minimum disclosure requirements of the AAPOR Code of Professional Ethics and Practice, every survey researcher should disclose each of the following elements in any report that is for public release, or be prepared to disclose this information promptly:

Name of the survey sponsor: The authors and affiliations are given in the MS. The funder was NIH IRP and that is acknowledge in the MS.

Name of the organization that conducted the survey: GfK

The exact wording of the questions being released: See above Supplement 2 for the survey instrument.

A definition of the population under study. What population is the survey designed to represent?: Adults in the United States.

A description of the sampling frame used to represent this population: The attached Methodology memo from GfK gives the technical details which follows this checklist below.

An explanation of how the respondents to the survey were selected: Random selection by computer. See GfK memo.

The total sample size: The total number of the invited participants and response rates and described in the MS, and in the GfK memo.

The method or mode of data collection: Internet survey

The dates and location of data collection: Collected in two waves between February 23, 2018 and April 03, 2018.

Estimates of sampling error, if appropriate: n/a.

A description of how the data were weighted (or a statement that they were not weighted), and any estimating procedures used to produce the final results: This is described in the MS; a more detailed description is given in the GfK memo below.

If the survey reports findings based on parts of the sample rather than the total sample, then the size of the subgroups reported should be disclosed: This is done throughout the MS.

GfK KnowledgePanel and Government & Academic Omnibus Methodology

Introduction

The GfK Group (formerly Knowledge Networks) is passionate about social science, health, and public policy research. We collaborate closely with client teams throughout the research process, while applying rigor in every step. We specialize in innovative online research that consistently gives leaders in academia, government, and business the confidence to make important decisions. GfK delivers affordable, statistically valid online research through KnowledgePanel® and leverages a variety of other assets, such as world-class advanced analytics, an industry-leading physician panel, an innovative platform for measuring online ad effectiveness, and a research-ready behavioral database of frequent supermarket and drug store shoppers.

GfK has recruited the first online research panel that is representative of the entire U.S. population. Panel members are randomly recruited through probability-based sampling, and households are provided with access to the Internet and hardware if needed.

GfK recruits panel members by using address-based sampling (ABS) methods (previously GfK relied on random-digit dialing [RDD] methods). Once household members are recruited for the panel and assigned to a study sample, they are notified by email for survey taking, or panelists can visit their online member page for survey taking (instead of being contacted by telephone or postal mail). This allows surveys to be fielded quickly and economically. In addition, this approach reduces the burden placed on respondents, since email notification is less intrusive than telephone calls and most respondents find answering online questionnaires more interesting and engaging than being questioned by a telephone interviewer. Furthermore, respondents have the convenience to choose what day and time to complete their assigned survey.

KnowledgePanel Methodology Information

KnowledgePanel is the largest online panel that relies on probability-based sampling techniques for recruitment; hence, it is the largest national sampling frame from which fully representative samples can be generated to produce statistically valid inferences for study populations. Our panel provides samples with the highest level of representativeness available in online research for measurement of public opinions, attitudes, and behaviors. The panel was first developed in 1999 by Knowledge Networks, a GfK company. Panel members are randomly selected so that survey results can properly represent the U.S. population with a measurable level of accuracy, features that are not obtainable from nonprobability panels (for comparisons of results from probability versus nonprobability methods, see Yeager et al., 2011[1]).

KnowledgePanel's recruitment process was originally based exclusively on a national RDD sampling methodology. In 2009, in light of the growing proportion of cellphone-only households, GfK migrated to an ABS recruitment methodology via the U.S. Postal Service's Delivery Sequence File (DSF). ABS not only improves population coverage, but also provides a more effective means for recruiting hard-to-reach individuals, such as young adults and minorities. Households without Internet connection are provided with a web-enabled device and free Internet service.

After initially accepting the invitation to join the panel, participants are asked to complete a short demographic survey (the initial Core Profile Survey); answers to this survey allow

---

[1] Yeager, D., Krosnick, J., Chang, L., Javitz, H., Levendusky, M., Simper, A. and R. Wang (2011). "Comparing the Accuracy of RDD Telephone Surveys and Internet Surveys Conducted With Probability and Non-Probability Samples." *Public Opinion Quarterly,* Winter 2011.

efficient panel sampling and weighting for future surveys. Upon completing the Core Profile Survey, participants become active panel members. All panel members are provided privacy and confidentiality protections.

ABS Recruitment

To enhance the DSF-based sampling frame used for address selection, we have various ancillary data appended to each household address, thus facilitating complex stratification plans.[2] Taking advantage of such refinements, quarterly samples are selected using a disproportionate stratified sampling methodology across four strata to address differential attrition rates:

1. Hispanic households with at least one 18 to 24 year-old
2. Remaining Hispanic households
3. Remaining households with at least one 18 to 24 year-old
4. All remaining households

Adults from sampled households are invited to join KnowledgePanel through a series of mailings, including an initial invitation letter, a reminder postcard, and a subsequent follow-up letter. Moreover, telephone refusal-conversion calls are made to nonresponding households for which a telephone number could be matched to a physical address. Invited households can join the panel by:

- Completing and mailing back a paper form in a postage-paid envelope
- Calling a toll-free hotline phone number maintained by GfK
- Going to a designated GfK website and completing the recruitment form online

Household Member Recruitment

During the initial recruitment survey, all household members are enumerated. Following enumeration, attempts are made to recruit every household member who is at least 13 years old to participate in KnowledgePanel surveys. For household members aged 13 to 17, consent is collected from the parents or the legal guardian during the initial recruitment interview. No direct communication with teenagers is attempted before obtaining parental consent. While surveys can be conducted with these teens directly, in most instances teen surveys are conducted by first selecting a sample of active members who are parents. This parent route alternative makes it possible to reach a larger sample of teens.

Survey Sampling from KnowledgePanel

Once panel members are recruited and profiled by completing our Core Profile Survey, they become eligible for selection for client surveys. Typically, specific survey samples are based on the equal probability selection method (EPSEM) for general population surveys. Customized stratified random sampling based on “profile” data can also be implemented as required by the study design. Profile data can also be used when a survey calls for pre-screening—that is, members are drawn from a subsample of the panel, such as females, Republicans, grocery shoppers, etc. (This can reduce screening costs, particularly for rare subgroups.) In such cases, we take care to ensure that all subsequent survey samples drawn that week are selected in such a way as to result in a sample that remains representative of the panel distributions.

---

[2] Fahimi, M. and D. Kulp (2009). “Address-Based Sampling – Alternatives for Surveys That Require Contacts with Representative Samples of Households.” *Quirk’s Marketing Research Review*, May 2009.

Survey Administration

Once assigned to a survey, members receive a notification email letting them know there is a new survey available for them to complete. This email notification contains a link that sends them to the survey. No login name or password is required. The field period depends on the client's needs and can range anywhere from a few hours to several weeks.

After three days, automatic email reminders are sent to all non-responding panel members in the sample. Additional email reminders are sent as needed. To assist panel members with their survey taking, each individual has a personalized member portal listing all assigned surveys that have yet to be completed.

GfK also operates an ongoing modest incentive program to encourage participation and create member loyalty. The incentive program includes special raffles and sweepstakes with both cash rewards and other prizes to be won. Typically, we assign panel members no more than one survey per week. On average, panel members complete two to three surveys per month with durations of 10 to 15 minutes per survey. An additional incentive is usually provided for longer surveys.

Response Rates

As a member of the American Association of Public Opinion Research (AAPOR), GfK follows the AAPOR standards for response rate reporting. While the AAPOR standards were established for single survey administrations and not for multi-stage panel surveys, we use the Callegaro-DiSogra (2008)[3] algorithms for calculating KnowledgePanel survey response rates. Generally, the KnowledgePanel survey completion rate is about 60%, with minor variations due to survey length, topic, sample specifications, and other fielding characteristics. In contrast, virtually all surveys that employ nonprobability online panels typically achieve survey completion rates in the low single digits.

Government & Academic Omnibus Survey Completion Rates

The field period and completion and qualification rates for this Government & Academic Omnibus survey are presented below:

Field Start	Field End	N Fielded	N Completed	Completion Rate	N Qualified	Qualification Rate
02/23/2018	03/06/2018	1759	1021	58%	1021	100%

* Of the qualified cases, 20 were removed during data cleaning, leaving 1001 cases in the final deliverable.

Field Start	Field End	N Fielded	N Completed	Completion Rate	N Qualified	Qualification Rate
03/23/18	04/03/18	1980	1021	52%	1021	100%

* Of the qualified cases, 18 were removed during data cleaning, leaving 1003 cases in the final deliverable.

Sample Weighting

As detailed above, significant resources and infrastructure are devoted to the recruitment process for KnowledgePanel so that our active panel members can properly represent the adult population of the U.S. This representation is achieved not only with respect to a broad set of geodemographic indicators, but also for hard-to-reach adults (such as those without Internet access or Spanish-language-dominant Hispanics) who are recruited in proper proportions. Consequently, the raw distribution of KnowledgePanel mirrors that of the U.S.

---

[3] Callegaro, M. and C. DiSogra (2008). "Computing Response Metrics for Online Panels." *Public Opinion Quarterly*, Vol. 72, No. 5.

adults fairly closely, barring occasional disparities that may emerge for certain subgroups due to differential attrition.

For selection of general population samples from KnowledgePanel, a patented methodology has been developed that ensures all samples behave as EPSEM samples. Briefly, this methodology starts by weighting the pool of active members to the geodemographic benchmarks secured from the latest March supplement of the U.S. Census Bureau's Current Population Survey (CPS) along several dimensions. Using the resulting weights as measures of size, a probability-proportional-to-size (PPS) procedure is used to select study specific samples. It is the application of this PPS methodology with the imposed size measures that produces fully self-weighing samples from KnowledgePanel, for which each sample member can carry a design weight of unity. Moreover, in instances where a study design requires any form of oversampling of certain subgroups, such departures from an EPSEM design are accounted for by adjusting the design weights in reference to the CPS benchmarks for the population of interest.

Study-Specific Final Weights

Once all survey data have been collected and processed, design weights are adjusted to account for any differential nonresponse that may have occurred. Depending on the specific target population for a given study, geodemographic distributions for the corresponding population are obtained from the CPS, the U.S. Census Bureau's American Community Survey (ACS), or in certain instances from the weighted KnowledgePanel profile data. For this purpose an iterative proportional fitting (raking) procedure is used to produce the final weights. In the final step, calculated weights are examined to identify and, if necessary, trim outliers at the extreme upper and lower tails of the weight distribution. The resulting weights are then scaled to aggregate to the total sample size of all eligible respondents.

The following benchmark distributions were used for the raking adjustment of the design weights for respondents who completed this Government & Academic Omnibus survey:

- Gender (Male/Female)
- Age (18–29, 30–44, 45–59, and 60+)
- Race/Hispanic Ethnicity (White/Non-Hispanic, Black/Non-Hispanic, Other/Non-Hispanic, 2+ Races/Non-Hispanic, Hispanic)
- Education (Less than High School, High School, Some College, Bachelor and beyond)
- Census Region (Northeast, Midwest, South, West)
- Household Income ($0-$25K, $25-$49,999, $50K-$74,999, $75K-$99,999, $100K-$149,999, $150K+)
- Metropolitan Area (Yes, No)
